# Impact of excessive daytime sleepiness on the progression of freezing of gait in *de novo* Parkinson’s disease: a cohort study

**DOI:** 10.1007/s10072-024-07738-8

**Published:** 2024-09-26

**Authors:** Min Chen, Yanjie Guo, Xuewei Zhang, Maoyun Zhao, Tinghua Zheng, Jingyang Song, Feng-Tao Liu, Hongxia Xing

**Affiliations:** 1 Xinxiang Medical University, Xinxiang, 453003 Henan China; 2 Key Laboratory of Movement Disorders, Xinxiang, 453000 Henan China; 3 Department of Neurology, The Third Affiliated Hospital of Xinxiang Medical University, Xinxiang, 453000 Henan China; 4 Department of Neurology, Huashan Hospital North, Fudan University, Shanghai, 200040 China; 5 Institute of Rehabilitation, Xinxiang Medical University, Xinxiang, 453003 Henan China; 6 Department of Rehabilitation, The Third Affiliated Hospital of Xinxiang Medical University, Xinxiang, 453000 Henan China

**Keywords:** Parkinson’s disease, Freezing of gait, Excessive daytime sleepiness, Sleep disorders

## Abstract

**Background:**

Excessive daytime sleepiness (EDS) and freezing of gait (FOG) are prevalent non-motor and motor symptoms in patients with Parkinson’s disease (PD), significantly impacting their quality of life. However, the correlation between EDS and FOG progression in *de novo* PD patients remains controversial.

**Methods:**

A total of 328 participants from the Parkinson’s Progression Markers Initiative (PPMI) were divided into two groups: 43 with EDS (EDS group) and 285 without EDS (nEDS group). The cumulative incidence of FOG was assessed at the 5-year follow-up using Kaplan–Meier and log-rank tests. Multivariate Cox proportional hazards models were used to assess the impact of EDS on FOG progression in PD patients, with validation for robustness through sensitivity and subgroup analyses.

**Results:**

The EDS group experienced a higher incidence of FOG throughout the 5-year follow-up than did the nEDS group. Multivariate Cox proportional hazards models showed significantly association between EDS severity and enhanced risk of developing FOG (HR = 1.076, 95% CI:1.007 ~ 1.149, *P* = 0.031). For sensitivity analysis, parallel analyses were performed by substituting the independent variable with categorical variables, which yielded analogous outcomes (HR = 1.837, 95% CI:1.063 ~ 3.174, *P* = 0.029). Furthermore, subgroup analyses based on sex, age, TD/PIGD classification, depressive symptoms, cognitive impairment, mean caudate nucleus uptake level, mean putamen nucleus uptake level and CSF Aβ-42 level revealed no significant interactions between subgroups (all *P* values for interaction were > 0.05).

**Conclusion:**

EDS is a potential prognosis factor for the progression of FOG in patients with PD.

**Supplementary Information:**

The online version contains supplementary material available at 10.1007/s10072-024-07738-8.

## Introduction

As one of the prominent motor symptoms in Parkinson’s disease (PD), freezing of gait (FOG) manifests as an abrupt and transient cessation in gait, which is often described subjectively as a sensation akin of the feet glued to the ground [1]. It is typically triggered at gait initiation, turns, approaches to obstacles, or ambulation, severely impairing the mobility function [2]. Previous research indicated a high FOG prevalence in PD, as approximately a quarter of early PD patients experiencing FOG [3], and more than half of the patients suffer from it in the latter stages [4]. FOG increases the incidence of falls and injuries in patients with PD, deprives their mobility and functional independence [5], and greatly impacts their life quality [4]. Therefore, identifying and managing FOG timely during clinical practice is of vital importance.

The detailed mechanisms of FOG are complex and have not been clearly elucidated, and a range of studies have explored the potential risk factors to it, including sleep disturbances [5]. Excessive daytime sleepiness (EDS) is a common non-motor symptom in PD, which refers to the condition that a patient cannot stay awake or conscious during the daytime, with inappropriate and uncontrolled episodes of sleep onset [6]. Up to 60% of PD patients might experience EDS, and it can also be induced by the application of dopamine agonists [7]. Some studies have ever explored the association between EDS and FOG in PD, but the results are still inconsistent. Bank et al. [8] declared EDS a risk factor for the development of FOG, while Kim et al. [9] reported that the Epworth sleepiness scale (ESS) scores correlated positively with FOG, though the correlation did not survive after adjustment. Although some studies preferred EDS in PD patients with FOG [10–12], others found no differences in ESS scores between PD patients with and without FOG [13–15]. However, limited research has explored the impact of EDS on FOG progression in untreated PD patients during longitudinal follow-up.

In this study, we explored the effects of EDS on the development of FOG in the patients with PD in a *de novo* cohort from the Parkinson’s Disease Progression Marker Initiative (PPMI). We wish to provide solid evidence on the recognition and intervention of FOG in PD.

## Materials and methods

### Study participants

The study participants and related data were acquired from the PPMI database on 3/1/2024. PPMI, established by the Michael J. Fox Foundation, is an international, prospective, observational, multinational and multicenter study that aims to recruit a large sample of subjects to search for clinical biomarkers of PD onset and progression [16]. Further protocol details and research methods are accessible at http://www.ppmi-info.org/study-design. Before the recruitment began, each participant signed an informed consent form. The study received approval from the institutional review board at each PPMI site. The registration number for clinicaltrials.gov was NCT01141023. All patients received structured follow-ups after enrollment. In patients who withdrew or commenced symptomatic treatment, follow-up sessions were arranged before the scheduled visit.

**Fig. 1:**
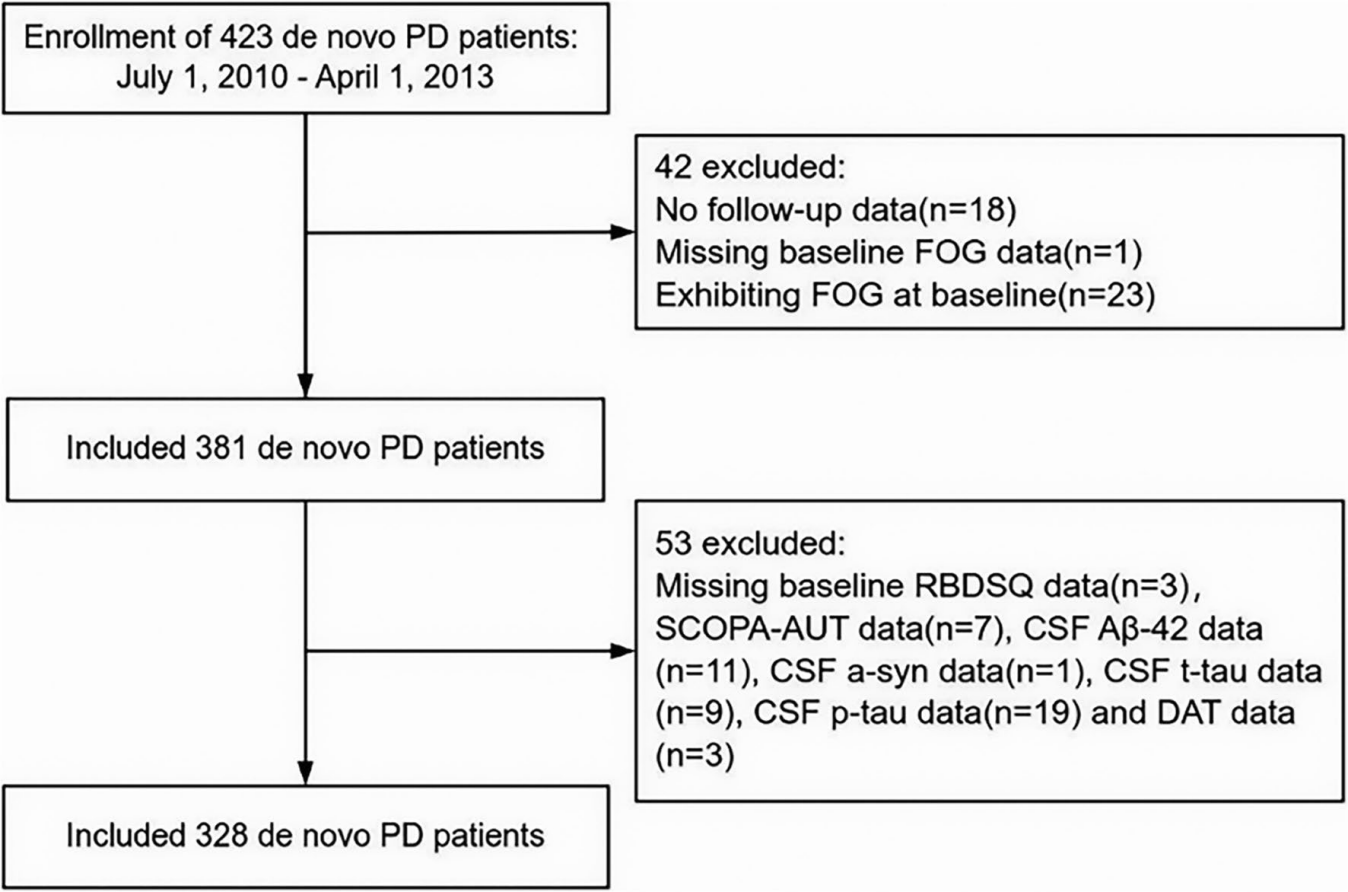
Flowchart of participant selection

From July 1, 2010, to April 1, 2013, there were 423 *de novo* PD patients recruited in the PPMI cohort. Data up to a 5-year follow-up were included in this study. Patients without FOG at baseline but underwent detailed evaluations of FOG and critical clinical covariates (including TD/PIGD classification, motor function scale scores, emotional and cognitive scale scores, sleep scale scores, autonomic function scale scores, activities of daily living scale scores, striatal dopamine transporter protein (DAT) uptake and cerebrospinal fluid (CSF) indicators) at baseline and 5-year follow-up were included in this study. Finally, 328 patients were included in the current analysis. The flowchart for participants selection is presented in Fig. 1.

### Clinical assessments

FOG was assessed by the item 2.13 (freezing) and item 3.11 (FOG) of the Movement Disorders Society Unified Parkinson’s Disease Rating Scale (MDS–UPDRS) [17]. If one or both of the scores were ≥ 1 throughout the follow-up [18], FOG was considered to have occurred. FOG severity was defined as the total score of MDS–UPDRS item 2.13 and 3.11 [9].The severity of EDS was evaluated by the ESS scales, with a score ranging from 0 to 24. The higher the total ESS score was, the more severe the EDS presented. An ESS score of 10 or higher indicated the existence of EDS [19].

The other clinical variables collected in the study included age, sex, years of education, disease duration, age at onset, and MDS-UPDRS (comprising Part I nonmotor symptoms during daily life, Part II motor symptoms during daily life, and Part III motor examination) [17]. Olfactory function was tested by the University of Pennsylvania Smell Identification Test (UPSIT). Functional dependence was assessed by the Modified Schwab & England Activities of Daily Living (MSE-ADL) score. Cognitive functions and the symptoms of depression and anxiety were measured using the Montreal Cognitive Assessment (MoCA), the Geriatric Depression Scale (GDS), and the State-Trait Anxiety Inventory (STAI). The severity of RBD was assessed by the REM Behavioral Sleep Disorders Questionnaire (RBDSQ), and the autonomic functions were assessed by the Autonomic Outcome Scale for PD (SCOPA-AUT).

The biomarkers collected in our study included DAT SPECT imaging, CSF amyloid-β (Aβ-42), tau and α-synuclein proteins assessments. The DAT SPECT imaging followed the PPMI protocol and adhered to standard operating procedures. Finally, the mean DAT binding values in the caudate and putamen were calculated and included. Standardized lumbar puncture approaches were used to obtain CSF [20]. The CSF biomarkers, including Aβ-42, total tau (t-tau), and phosphorylated tau (p-tau), were evaluated using the xMAP-Luminex platform. The levels of CSF α-synuclein were determined using an ELISA kit that was commercially available (Covance, Dedham, MA). The detailed protocol information could be obtained from https://www.ppmi-info.org/study-design/research-documents-and-sops.

### Statistical analysis

Normally distributed continuous variables were expressed as mean ± standard deviation (SD), whereas skewed data were expressed as median with the 25th and the 75th percentiles (*P*_25_, *P*_75_). Continuous variables were compared between groups using Student’s t-test or Mann–Whitney U test, based on whether the data were normally distributed. Categorical data were compared using Fisher’s exact or chi-square test and expressed as a proportion (%). The cumulative incidence of FOG in the EDS and nEDS groups was compared using Kaplan–Meier and log-rank analyses. Multivariate Cox proportional hazards models, adjusting for potential confounders, were used to analyze the association between EDS and FOG progression. Hazard ratios (HRs) and 95% confidence intervals (CIs) were subsequently calculated. Before constructing the multivariate model, the generalized variance inflation factor (GVIF) was computed to determine the collinearity between EDS severity and other variables. Variables with GVIF^(1/2Df) < 2 were included. The selection of covariates was based on the following principles: (1) variables ever reported in prior researches, e.g. age and sex; (2) variables that have great effects to the model, introducing or removing which from the basic or full model could induce the hazard ratio changes exceeding 10%, and (3) variables that exhibit a significance level of *P* < 0.05 in univariate analysis. Following the criteria above, multiple adjustment models were developed: Model I was adjusted for age and sex. Model II was adjusted for age, sex, disease duration, TD/PIGD classification, MDS-UPDRS I, MDS-UPDRS II, SCOPA-AUT, and CSF Aβ-42. Model III was further adjusted for GDS, STAI, MoCA, MSE-ADL, mean caudate uptake, and mean putamen uptake.

Furthermore, subgroup analysis was also carried out to explore whether the included variables (age, sex, TD/PIGD classification, depression, cognitive impairment, striatal DAT uptake, and CSF Aβ-42 levels) could affect the association with FOG. Interactions between the FOG status and the severity of EDS, as well as other variables mentioned above, were examined separately. Likelihood-ratio tests were used to inspect the modifications and interactions of subgroups.

As the proportion of missing values for all covariates in our dataset was less than 5%, we have opted to exclude samples with missing data to ensure the integrity and accuracy of our analysis. All calculations were undertaken with R 4.3.2 (http://www.R-project.org, The R Foundation) and Free Statistics software version 1.9.1. A statistically significant difference was considered for a two-sided *P* < 0.05.

## Results

### Demographic information and clinical characteristics

A total of 328 patients with *de novo* PD were included in the analysis, including 43 patients with EDS (EDS group) and 285 without EDS (nEDS group). The baseline characteristics of the EDS and nEDS groups were displayed in Table 1. There was no discernible difference in terms of age, sex, age of onset, disease duration, MDS-UPDRS III score, striatal DAT bindings ratio and CSF biomarkers between the EDS group and nEDS group. However, the patients in the EDS group had longer years of education (*P* = 0.036), more severe MDS-UPDRS II score (*P* < 0.001) as well as other non-motor symptoms, notably, higher ESS score (*P* < 0.001), higher MDS-UPDRS I score (*P* = 0.017), total SCOPA-AUT scores (*P* < 0.001) and GDS scores (*P* = 0.036).

**Table 1:** Demographic information and clinical characteristics in patients with *de novo* PD associated with different EDS statuses

Clinical variables	Total (*n* = 328)	EDS (*n* = 43)	nEDS (*n* = 285)	*P* value
Age	62.0 ± 9.7	63.7 ± 8.8	61.8 ± 9.8	0.226
Sex (M/F)	215/113	28/15	187/98	0.949
Education (years)	15.5 ± 2.9	16.4 ± 2.5	15.4 ± 3.0	**0.036**
Age of onset (year)	60.0 ± 9.9	61.9 ± 8.6	59.8 ± 10.1	0.196
Disease duration (month)	4.2 (2.5, 7.6)	5.3 (2.8, 10.8)	4.0 (2.5, 7.1)	0.051
TD/PIGD classification, n (%)				0.278
TD	236 (72.0)	27 (62.8)	209 (73.3)	
PIGD	55 (16.8)	9 (20.9)	46 (16.1)	
Indeterminate	37 (11.3)	7 (16.3)	30 (10.5)	
MDS-UPDRS I	5.3 ± 3.7	6.5 ± 3.9	5.1 ± 3.6	**0.017**
MDS-UPDRS II	5.5 ± 3.9	7.5 ± 4.8	5.2 ± 3.6	** < 0.001**
MDS-UPDRS III	20.4 ± 8.5	21.7 ± 8.7	20.2 ± 8.5	0.266
MoCA	27.0 ± 2.3	27.0 ± 3.0	27.1 ± 2.2	0.938
GDS	2.0 (1.0, 3.0)	2.0 (1.0, 3.0)	1.0 (1.0, 3.0)	**0.035**
STAI	63.9 ± 17.6	67.2 ± 18.2	63.4 ± 17.5	0.184
ESS	5.0 (3.0, 8.0)	11.0 (10.0, 13.0)	5.0 (3.0, 6.0)	** < 0.001**
RBDSQ	3.0 (2.0, 5.0)	4.0 (2.5, 6.0)	3.0 (2.0, 5.0)	0.093
SCOPA-AUT	8.0 (5.0, 12.0)	11.0 (8.0, 15.0)	8.0 (5.0, 12.0)	** < 0.001**
SCOPA-AUT Cardiovascular	0 (0, 1.0)	0 (0, 1.0)	0 (0, 1.0)	0.129
UPSIT	22.2 ± 8.3	21.7 ± 9.0	22.2 ± 8.2	0.717
MSE-ADL	93.5 ± 5.8	93.4 ± 6.9	93.5 ± 5.6	0.885
DAT imaging (striatal binding ratio)				
Mean caudate uptake	2.0 ± 0.5	1.9 ± 0.5	2.1 ± 0.5	0.136
Mean putamen uptake	0.8 ± 0.3	0.8 ± 0.3	0.8 ± 0.3	0.321
CSF biomarkers				
Aβ-42, pg/mL	956.2 ± 415.5	917.0 ± 411.2	962.1 ± 416.5	0.508
a-syn, pg/mL	1573.8 ± 656.8	1652.0 ± 694.7	1562.0 ± 651.3	0.403
t-tau, pg/mL	173.7 ± 54.6	184.2 ± 57.1	172.1 ± 54.1	0.176
p-tau, pg/mL	14.8 ± 5.2	16.0 ± 6.0	14.7 ± 5.1	0.109

Data are shown as mean ± standard deviation, median (*P*_25_, *P*_75_), or prevalence (%). The *P* value in bold indicates *P* < 0.05, which indicates a significant difference between groups

*nEDS* Patients without EDS, *EDS* Patients with EDS, *TD* Tremor-Dominant, *PIGD* postural instability gait difficulty, *MDS-UPDRS I* Movement Disorders Society Unified Parkinson’s Disease Rating Scale part 1, *MDS-UPDRS II* Movement Disorders Society Unified Parkinson’s Disease Rating Scale part 2, *MDS-UPDRS III* Movement Disorders Society Unified Parkinson’s Disease Rating Scale part 3, *MoCA* Montreal Cognitive Assessment, *GDS* Geriatric Depression Scale, *STAI* State–Trait Anxiety Inventory, *ESS* Epworth sleepiness scale, *RBDSQ* REM Sleep Behavior Disorder Screening Questionnaire, *SCOPA-AUT* Scale for Outcomes in Parkinson’s Disease Autonomic, *SCOPA-AUT* Cardiovascular, SCOPA-AUT Cardiovascular Subscore, *UPSIT* University of Pennsylvania Smell Identification Test, *MSE-ADL* Modified Schwab & England Activities of Daily Living Scale, *DAT* Dopamine Transporter, *CSF* cerebrospinal fluid, *Aβ-42* amyloid β–42, *α-syn* total alpha-synuclein, *t-tau* total tau, *p-tau* phosphorylated tau

### The impact of EDS on the development of FOG

During the 5-year follow-up, up to 87 (26.6%) patients with PD ultimately developed FOG. In the EDS group, 20 (46.5%) patients turned to have FOG, while the frequency was 23.5% in the nEDS group. Additionally, among those FOG converters, 20 patients were positive in the baseline EDS status, while 67 patients were negative in the EDS assessment. A significantly higher proportion of EDS (20/87) was also found in the FOG converters, compared to that (23/241) in the FOG non-converters. Moreover, at baseline, the FOG converters exhibited higher ESS scores, older age and older age of onset, a higher proportion of PIGD subtypes, higher scores on the MDS-UPDRS I and II, higher GDS scores, STAI scores and SCOPA-AUT scores, lower MoCA scores and MSE-ADL scores, decreased striatal DAT uptake, and lower CSF Aβ-42 levels (as shown in Supplementary Table 1).

**Fig. 2:**
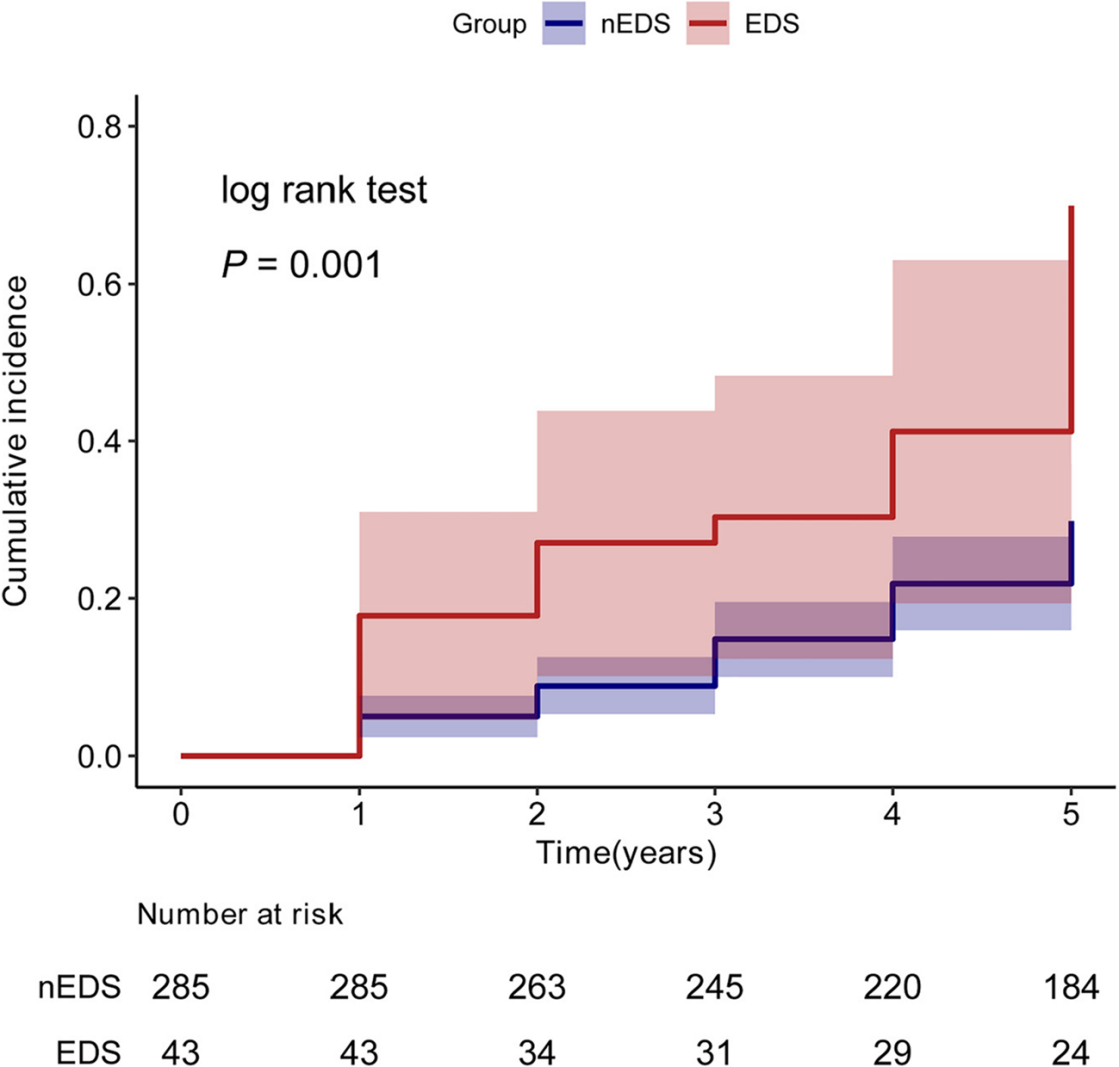
Associations between EDS with FOG progression in patients with PD. Kaplan–Meier estimates showed the EDS group had a higher risk of FOG progression (log-rank test, *P* = 0.001). EDS, excessive daytime sleepiness

According to Kaplan–Meier estimates, the cumulative incidence of FOG was significantly higher in the EDS group than that in the nEDS group (log-rank test, *P* = 0.001, Fig. 2). The effect of EDS on the incidence of FOG was explored by multifactorial Cox proportional hazards models. The results of the covariate screening were shown in the Supplementary Tables 1 and 2. The robust statistical results across all models were presented in Table 2.

**Table 2:** Results of the multivariable Cox proportional-hazards analysis for the predictors of FOG

Models	EDS severity	EDS status	
		nEDS	EDS
Crude model			
HR (95%CI)	1.121(1.059 ~ 1.186)	1(Ref)	2.308 (1.400 ~ 3.804)
*P* value	< 0.001		0.001
Model I			
HR (95%CI)	1.116(1.055 ~ 1.180)	1(Ref)	2.248 (1.363 ~ 3.708)
*P* value	< 0.001		0.002
Model II			
HR (95%CI)	1.077 (1.010 ~ 1.148)	1(Ref)	1.910 (1.125 ~ 3.244)
*P* value	0.024		0.017
Model III			
HR (95%CI)	1.076 (1.007 ~ 1.149)	1(Ref)	1.837 (1.063 ~ 3.174)
*P* value	0.031		0.029

Results are shown as hazard ratios (HRs) with 95% confidence intervals (CIs). *EDS* Excessive daytime sleepiness

Model 1: adjusted for age and sex

Model 2: adjusted Model 1 + disease duration, TD/PIGD classification, MDS-UPDRS I, MDS-UPDRS II, SCOPA-AUT and CSF Aβ-42

Model 3: adjusted Model 2 + GDS, STAI, MoCA, MSE-ADL, mean caudate uptake and mean putamen uptake

Specifically, in the unadjusted model (Crude model), EDS severity correlated positively with FOG progression in *de novo* PD patients, with each unit increase in EDS severity associated with a 12.1% elevated risk of developing FOG (HR = 1.121, 95% CI: 1.059 ~ 1.186, *P* < 0.001). In Model I, the risk of FOG increased by 11.6% for each unit increase in EDS severity after adjusting for age and sex (HR = 1.116, 95% CI: 1.055 ~ 1.180, *P* < 0.001). In Model II, after adjusting for disease duration, TD/PIGD classification, MDS-UPDRS I, MDS-UPDRS II, SCOPA-AUT and CSF Aβ-42 based on Model I, each unit increase in EDS severity was associated with a 7.7% increase in the risk of FOG (HR = 1.077, 95% CI: 1.01 ~ 1.148, *P* = 0.024). In the fully adjusted model (Model III), which included adjustments for GDS, STAI, MoCA, MSE-ADL, mean caudate uptake and mean putamen uptake in addition to Models I and II, the risk of FOG increased by 7.6% for each unit increase in EDS severity (HR = 1.076, 95% CI: 1.007 ~ 1.149, *P* = 0.031). The dependent variable was redefined as EDS status for sensitivity analysis, and an additional multivariate Cox regression analysis was conducted. The EDS was proved to be persisted as a significant predictor of FOG development, even in the fully adjusted model (HR = 1.837, 95% CI: 1.063 ~ 3.174, *P* = 0.029).

### Impact of the confounding factors on the development of FOG

**Fig. 3:**
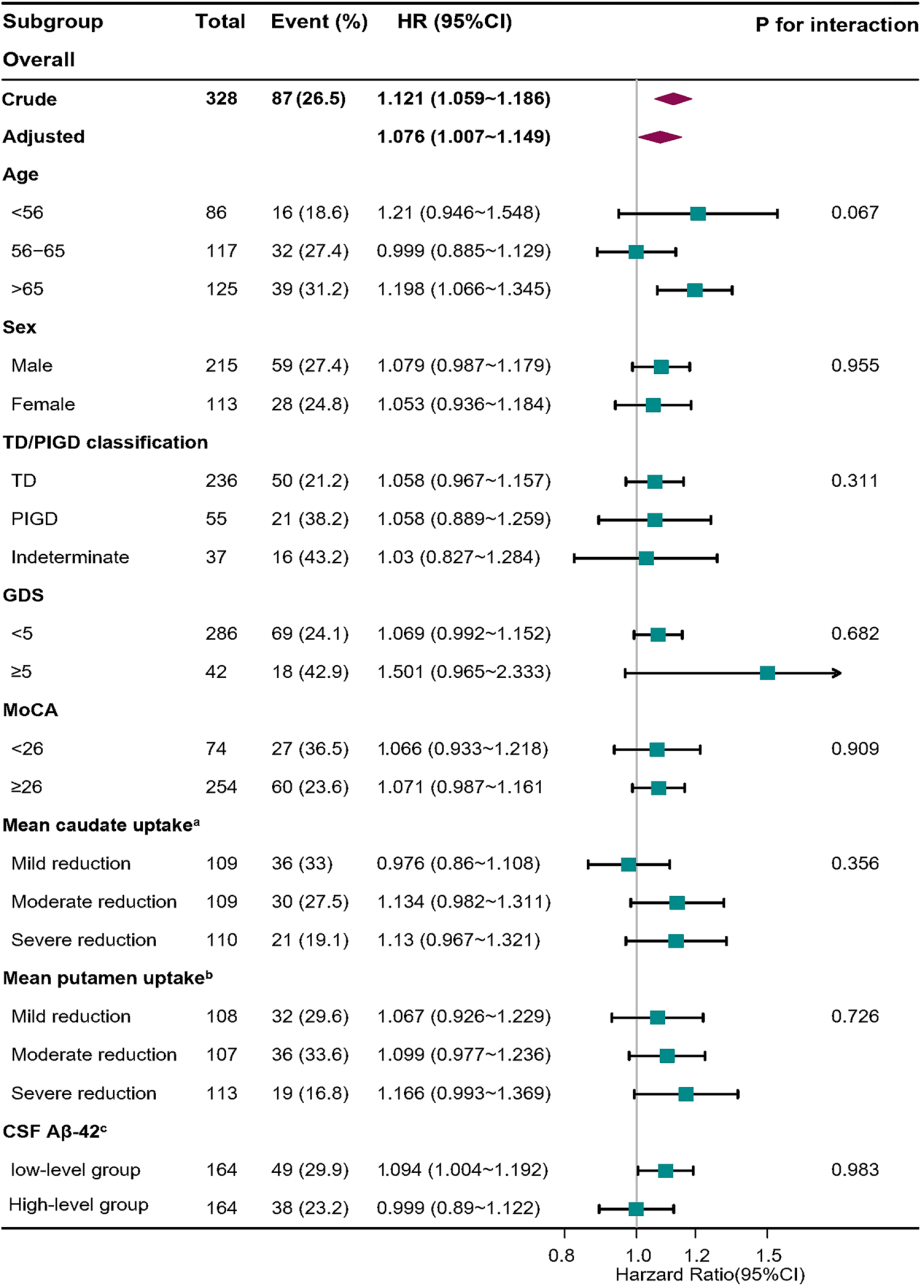
Forest plot of subgroup analysis on the impact of EDS severity on the progression of freezing of gait**.** Each stratification was adjusted for age, sex, disease duration, TD/PIGD classification, SCOPA-AUT, GDS, STAI, MOCA, MDS-UPDRS I, MDS-UPDRS II, MSE-ADL, mean caudate uptake, mean putamen uptake and CSF Aβ-42 except for the stratification factor itself**.** ^a^According to the baseline mean caudate uptake level, patients were divided into tertiles: mild reduction, moderate reduction, and severe reduction**.** ^b^According to the baseline mean putamen uptake level, patients were divided into tertiles: mild reduction, moderate reduction, and severe reduction**.** ^c^“Low-level” and “high-level” groups used the 50th percentile cutoff values for CSF Aβ42 levels (876.5 pg/ml)

Moreover, subgroup analyses were utilized to explore the impact of potential confounding factors, including sex, age, TD/PIGD classification, depression, cognitive impairment, mean caudate uptake levels, mean putamen uptake and CSF Aβ-42 levels. The results revealed similar risk estimates for FOG development across subgroups (all *P* values for interaction > 0.05, Fig. 3). Subgroup analysis using EDS status as the independent variable yielded similar outcomes (Supplementary Fig. 1). These findings suggest that the impact of EDS on FOG progression remained stable and unaffected by variations in covariates.

## Discussion

This study comprehensively investigated the relationship between baseline EDS and the development of FOG in *de novo* patients with PD by utilizing the 5-year longitudinal data from the PPMI. Our study substantiated baseline EDS as a strong prognostic predictor for the development of FOG in *de novo* PD patients, independent of the confounding factors of age, sex, disease duration, TD/PIGD classification, MDS-UPDRS I, MDS-UPDRS II, SCOPA-AUT, GDS, STAI, MoCA, MSE-ADL, striatum DAT uptake and CSF Aβ-42.

Previous studies have found that non-motor symptoms are closely related to motor symptoms in patients with PD, while non-motor symptoms tend to appear earlier than the onset of motor symptoms [21]. Therefore, non-motor symptoms may be predictive for recognizing motor symptoms such as FOG. EDS is an important non-motor symptom in drug-naive PD, but it is often neglected during clinical practice. In the current study, patients with EDS had higher MDS-UPDRS I and MDS-UPDRS II scores, more severe depressive symptoms, and autonomic dysfunction. According to Tholfsen et al. [22], daily sleepiness is common even among PD patients who have never taken medication. Yoo et al. [23] reported that patients with EDS in *de novo* PD had higher MDS-UPDRS II scores, higher MDS-UPDRS I nonmotor symptom scores, more nonmotor symptoms, and poorer health-related quality of life. The presence of more severe autonomic dysfunction and depressive symptoms in EDS patients was align with the previous study [24], which suggested the common pathogenesis of non-dopaminergic regulatory structures, such as the noradrenergic system. Dysfunction of the noradrenergic system has also been associated with the development of FOG [25]. Therefore, the non-drug-induced sleep disorders should receive serious attention, as they might provide some clues to the recognition of other disease related symptoms, such as FOG.

In this study, the cumulative incidence of FOG was higher in the EDS group than in the nEDS group after 5-year follow-up. Additionally, upon controlling for potential confounders, the analysis revealed the significant impact of EDS on the progression of FOG in patients with PD. Subgroup and sensitivity analyses further validated the robustness of these findings. This study confirms that EDS is a potential risk factor for FOG, which is consistent with previous research findings [8].

The underlying mechanisms might be related to the degeneration of structures involved in arousal within the brainstem, such as the locus coeruleus (LC) and the ascending reticular activating system, as previously reported [26]. Firstly, LC represents the principal cluster of noradrenergic neurons and plays a crucial role in the central nervous system. Dysfunction of the noradrenergic system has been associated with deficits in motor control, such as impaired balance, an increased incidence of falls, and the occurrence of FOG, and it is also correlated with nonmotor symptoms. Because dysfunction of the noradrenergic system may lead to decreased ability to regulate arousal and sleep cycles, triggering daytime sleepiness in patients [27]. In addition, methylphenidate influences the firing activity of the LC and can improve norepinephrine synaptic levels [28]. Some researchers found that methylphenidate was efficient in improving FOG as well as EDS in PD patients [29].

Moreover, the pathological commonality between EDS and FOG may primarily be related to the degeneration of cholinergic neurons connecting the pedunculopontine nucleus (PPN) and the thalamus. The PPN, a crucial component of the midbrain locomotor region and the reticular activating system, is interconnected with various areas, containing the cortex, basal ganglia, and thalamus. The PPN primarily comprises cholinergic, glutamatergic, and GABAergic neurons [30]. The PPN is involved in regulating gait and posture and modulating the sleep–wake cycle [31]. Davin et al. [32] found that low-frequency deep brain stimulation in the PPN region effectively improved EDS in nonhuman primate PD models. Previous preclinical trials also emphasized the effect of the PPN on movement [33], enabling the PPN to be a potential surgical target for deep brain stimulation (DBS) in treating FOG in PD patients. Meanwhile, sleep disorders increase the deposition of toxic substances such as Aβ, tau and α-synuclein in the brain, leading to progressive dysfunction of neural circuits and impacting patients’ gait [9, 34]. Therefore, although there is a clear correlation between daytime sleepiness and FOG, the specific pathological mechanisms need to be further explored in the future.

### Strengths and limitations

This study serves as a helpful reference for determining the associations between the risk of FOG and the severity and status of EDS. It utilizes up to 5 years of follow-up data from a well-designed PPMI cohort. In addition, various multifactorial Cox regression models, subgroup analyses, and sensitivity analyses were performed to verify that the results were stable and reliable. However, there are several limitations to this study. The primary limitation lies in its retrospective design, which is attempted to be mitigated by using Kaplan–Meier and log-rank methods to compare event accumulation curves. Secondly, the assessment of FOG and EDS was relied on relatively subjective methods, lacking objective clinical evidence, such as utilizing wearable devices, virtual reality devices, and polysomnography monitoring systems. Thirdly, considering the constrained sample size, the statistical power was enhanced by incorporating covariate control within the multivariate model. Despite the comprehensive adjustment for relevant potential confounders, residual confounding may persist, potentially leading to overestimated observed associations. Subsequent research should include broader target populations, more extended follow-up periods, more objective data, and prospective study designs to further explore the correlation between FOG and EDS and the underlying pathologic mechanisms behind it.

## Conclusion

In conclusion, EDS is an independent potential risk factor for the development of FOG in patients with PD. Understanding the connection between EDS and FOG in PD is expected to identify patients at risk for FOG early, facilitating the development of personalized interventions that target both symptoms.

## Supplementary Information

Below is the link to the electronic supplementary material.

Supplementary file1 (DOCX 486 KB)

## Data Availability

The data supporting the findings of this study are openly available in PPMI at https://www.ppmi-info.org.
