# Supplementary material for: Impact of excessive daytime sleepiness on the progression of freezing of gait in de novo Parkinson’s disease: a cohort study
Source: Neurol Sci. 2024 Sep 26;46(2):723–31. doi: 10.1007/s10072-024-07738-8 (PMC11772505; doi:10.1007/s10072-024-07738-8)
Supplement: Supplementary file 1 — Supplementary file1 (DOCX 486 KB) [file 10072_2024_7738_MOESM1_ESM.docx]

**Supplementary Table 1** Comparison of baseline demographic and clinical characteristics among patients who developed FOG and those who without the development of FOG

| Clinical variables | FOG converters  (n = 87) | FOG non-converters  (n = 241) | *P* value |
| --- | --- | --- | --- |
| EDS, n (%) | 20 (23) | 23 (9.5) | **0.001** |
| ESS score | 7.0 (4.0, 9.0) | 5.0 (3.0, 7.0) | **< 0.001** |
| FOG severity^a^ | 1.0 (1.0, 1.0)^b^ | 0 (0, 0) | **< 0.001** |
| Age | 63.9 ± 10.1 | 61.4 ± 9.4 | **0.038** |
| Sex (M/F) | 59/28 | 156/85 | 0.604 |
| Education(years) | 15.8 ± 3.3 | 15.5 ± 2.8 | 0.400 |
| Age of onset (year) | 62.2 ± 10.2 | 59.3 ± 9.7 | **0.019** |
| Disease duration(month) | 3.5 (2.2, 6.5) | 4.4 (2.6, 8.2) | 0.100 |
| TD/PIGD classification, n (%) |  |  | **0.002** |
| TD | 50 (57.5) | 186 (77.2) |  |
| PIGD | 21 (24.1) | 34 (14.1) |  |
| Indeterminate | 16 (18.4) | 21 (8.7) |  |
| MDS-UPDRS Ⅰ score | 6.6 ± 4.1 | 4.8 ± 3.4 | **< 0.001** |
| MDS-UPDRS Ⅱ score | 7.2 ± 3.9 | 4.8 ± 3.6 | **< 0.001** |
| MDS-UPDRS III score | 21.8 ± 8.1 | 19.9 ± 8.6 | 0.074 |
| MoCA score | 26.6 ± 2.7 | 27.2 ± 2.1 | **0.021** |
| GDS score | 2.0 (1.0, 4.0) | 1.0 (0.0, 3.0) | **< 0.001** |
| STAI score | 67.7 ± 18.8 | 62.5 ± 17.0 | **0.018** |
| RBDSQ score | 4.0 (2.0, 6.0) | 3.0 (2.0, 5.0) | 0.123 |
| SCOPA-AUT total score | 11.0 (6.0, 15.0) | 8.0 (5.0, 11.0) | **< 0.001** |
| SCOPA-AUT Cardiovascular | 0 (0 1.0) | 0 (0 1.0) | **0.003** |
| UPSIT score | 21.2 ± 9.1 | 22.5 ± 7.9 | 0.216 |
| MSE-ADL score | 92.4 ± 5.4 | 93.9 ± 5.9 | **0.042** |
| DAT imaging (striatal binding ratio) |  |  |  |
| Mean caudate uptake | 1.9 ± 0.5 | 2.1 ± 0.5 | **0.003** |
| Mean putamen uptake | 0.8 ± 0.3 | 0.9 ± 0.3 | **0.005** |
| CSF biomarkers |  |  |  |
| Aβ-42, pg/mL | 849.4 ± 324.9 | 994.8 ± 437.9 | **0.005** |
| a-syn, pg/mL | 1498.9 ± 571.4 | 1600.9 ± 684.1 | 0.215 |
| t-tau, pg/mL | 175.8 ± 55.3 | 172.9 ± 54.4 | 0.674 |
| p-tau, pg/mL | 15.1 ± 5.6 | 14.7 ± 5.1 | 0.612 |

Data are shown as mean ± standard deviation, median (*P*_25_, *P*_75_), or prevalence (%). The *P* value in bold means *P* <0.05 and we define them have significant difference between groups.

^a^ Total score for MDS-UPDRS items 2.13 and 3.11 at the time of the first occurrence of FOG.

^b^ n=86 due to missing data from one patient for item 3.11 in the fourth-year follow-up.

FOG, freezing of gait; nEDS, Patients without EDS; EDS, Patients with excessive daytime sleepiness; TD, Tremor-Dominant; PIGD, postural instability gait difficulty; MDS-UPDRS Ⅰ, Movement Disorders Society Unified Parkinson’s Disease Rating Scale part 1; MDS-UPDRS Ⅱ, Movement Disorders Society Unified Parkinson’s Disease Rating Scale part 2; MDS-UPDRS III, Movement Disorders Society Unified Parkinson’s Disease Rating Scale part 3; MoCA, Montreal Cognitive Assessment; GDS, Geriatric Depression Scale; STAI, State–Trait Anxiety Inventory; RBDSQ, REM Sleep Behavior Disorder Screening Questionnaire; SCOPA-AUT, Scale for Outcomes in Parkinson’s Disease Autonomic; SCOPA-AUT Cardiovascular, SCOPA-AUT Cardiovascular Subscore; UPSIT, University of Pennsylvania Smell Identification Test; MSE-ADL, Modified Schwab & England Activities of Daily Living Scale; DAT, Dopamine Transporter; CSF cerebrospinal fluid; Aβ-42, amyloid β–42; α-syn, total alpha-synuclein; t-tau, total tau; p-tau, phosphorylated tau

**Supplementary Table 2** Selection of covariates in *de novo* PD patients

|  | Forward selection | | Backward elimination | | |
| --- | --- | --- | --- | --- | --- |
|  | Coefficient | Change percentage | Coefficient | | Change percentage |
| Crude | 0.11 | Ref. | 0.07 | Ref. | |
| Age | 0.11 | -3.9 | 0.07 | 6.1 | |
| Sex | 0.11 | 0.1 | 0.07 | -0.1 | |
| Education | 0.12 | 2.6 | 0.06 | -5.1 | |
| Age of onset | 0.11 | -4.6 | 0.07 | 6.3 | |
| Disease duration | 0.13 | 9.9 | 0.04 | **-35.4** | |
| TD/PIGD classification | 0.1 | **-12.7** | 0.08 | **15** | |
| MDS-UPDRS Ⅰ score | 0.09 | **-19.8** | 0.07 | 6.1 | |
| MDS-UPDRS Ⅱ score | 0.08 | **-29.9** | 0.08 | **10.8** | |
| MDS-UPDRS III score | 0.11 | -4 | 0.07 | 0.4 | |
| MoCA score | 0.11 | -1.3 | 0.06 | -9.4 | |
| GDS score | 0.11 | -1.4 | 0.06 | -4.6 | |
| STAI score | 0.11 | -2.3 | 0.07 | -2 | |
| RBDSQ score | 0.11 | -6.2 | 0.07 | -3.8 | |
| SCOPA-AUT total score | 0.09 | **-20.8** | 0.07 | 5.3 | |
| UPSIT score | 0.11 | -0.8 | 0.07 | 3 | |
| MSE-ADL score | 0.11 | -5.7 | 0.07 | 0.8 | |
| Mean caudate uptake | 0.1 | -8.4 | 0.07 | 1 | |
| Mean putamen uptake | 0.11 | -7.8 | 0.07 | 0.6 | |
| CSF Aβ-42 | 0.11 | -4.8 | 0.08 | **14.5** | |
| CSF a-syn | 0.12 | 3 | 0.07 | -0.9 | |
| CSF t-tau | 0.11 | -0.2 | 0.06 | -4.9 | |
| CSF p-tau | 0.11 | -0.6 | 0.07 | -1.9 | |

Dependent variable: ESS score

The number in bold means a more than 10% change in the matched hazard ratio

TD, Tremor-Dominant; PIGD, postural instability gait difficulty; MDS-UPDRS Ⅰ, Movement Disorders Society Unified Parkinson’s Disease Rating Scale part 1; MDS-UPDRS Ⅱ, Movement Disorders Society Unified Parkinson’s Disease Rating Scale part 2; MDS-UPDRS III, Movement Disorders Society Unified Parkinson’s Disease Rating Scale part 3; MoCA, Montreal Cognitive Assessment; GDS, Geriatric Depression Scale; STAI, State–Trait Anxiety Inventory; RBDSQ, REM Sleep Behavior Disorder Screening Questionnaire; SCOPA-AUT, Scale for Outcomes in Parkinson’s Disease Autonomic; UPSIT, University of Pennsylvania Smell Identification Test; MSE-ADL, Modified Schwab & England Activities of Daily Living Scale; DAT, Dopamine Transporter; CSF cerebrospinal fluid; Aβ-42, amyloid β–42; α-syn, total alpha-synuclein; t-tau, total tau; p-tau, phosphorylated tau


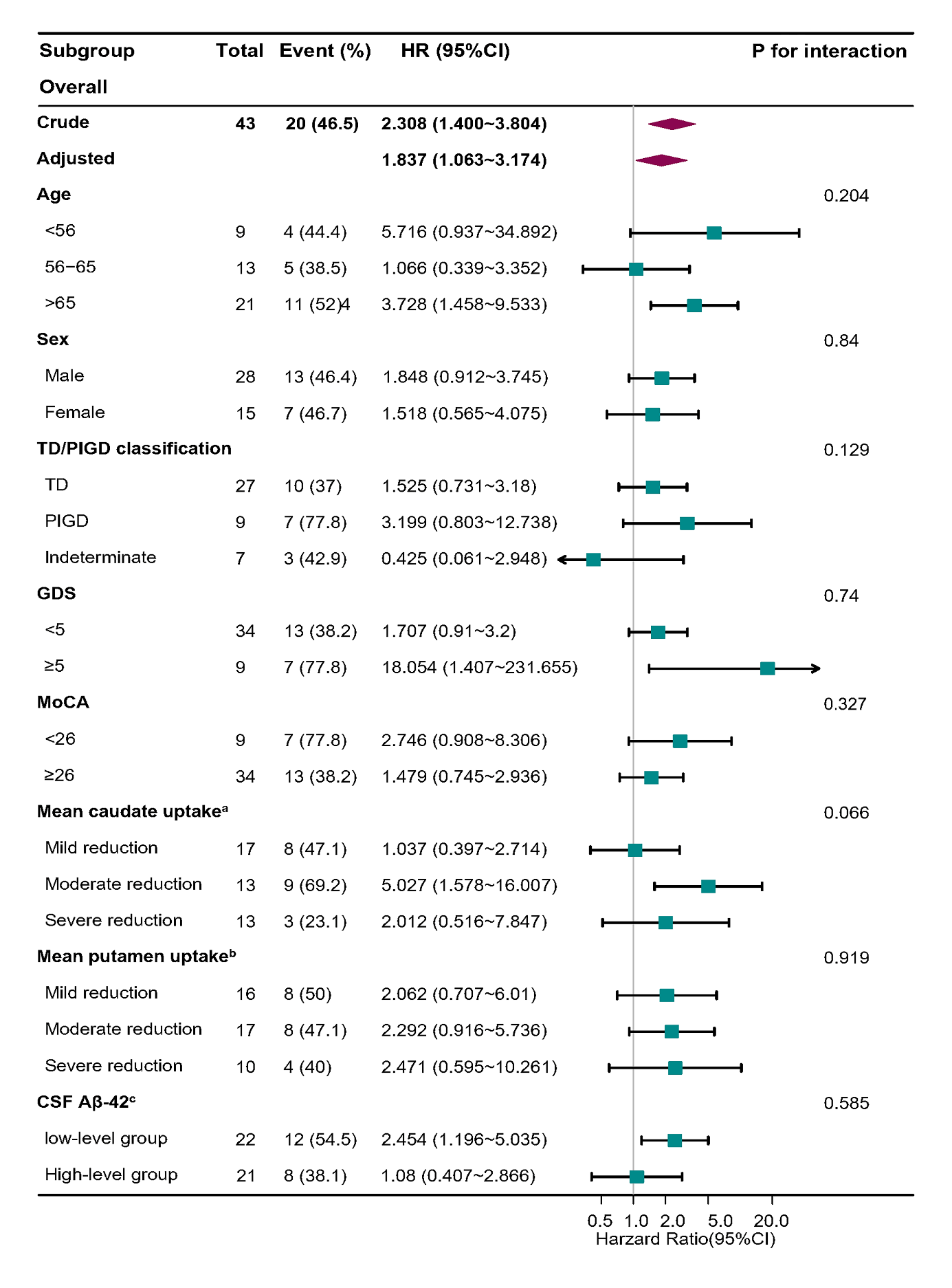


**Supplementary Fig. 1** Forest plot of subgroup analysis on the association between EDS status and the progression of freezing of gait

Each stratification was adjusted for age, sex, disease duration, TD/PIGD classification, SCOPA-AUT, GDS, STAI, MoCA, MDS-UPDRS Ⅰ, MDS-UPDRS Ⅱ, MSE-ADL, mean caudate uptake, mean putamen uptake and CSF Aβ-42 except the stratification factor itself

^a^According to the baseline mean caudate uptake level, patients were divided into tertiles: mild reduction, moderate reduction, and severe reduction

^b^According to the baseline mean putamen uptake level, patients were divided into tertiles: mild reduction, moderate reduction, and severe reduction

^c^“Low-level” and “high-level” groups used the 50th percentile cutoff values for CSF Aβ42 levels (876.5 pg/ml)
